# Paleopathological Study of Dwarfism-Related Skeletal Dysplasia in a Late Joseon Dynasty (South Korean) Population

**DOI:** 10.1371/journal.pone.0140901

**Published:** 2015-10-21

**Authors:** Eun Jin Woo, Won-Joon Lee, Kyung-Seok Hu, Jae Joon Hwang

**Affiliations:** 1 Department of Oral Biology, Division in Anatomy & Developmental Biology, BK21 PLUS Project, Yonsei University College of Dentistry, Seoul, Republic of Korea; 2 Institute of Forensic Science, Seoul National University College of Medicine, Seoul, Republic of Korea; 3 Department of Oral Biology, Division in Anatomy & Developmental Biology, Human Identification Research Institute, Yonsei University College of Dentistry, Seoul, Republic of Korea; 4 Department of Oral and Maxillofacial Radiology, Dental Hospital of Yonsei University College of Dentistry, Seoul, Republic of Korea; Ohio State University, UNITED STATES

## Abstract

Skeletal dysplasias related to genetic etiologies have rarely been reported for past populations. This report presents the skeletal characteristics of an individual with dwarfism-related skeletal dysplasia from South Korea. To assess abnormal deformities, morphological features, metric data, and computed tomography scans are analyzed. Differential diagnoses include achondroplasia or hypochondroplasia, chondrodysplasia, multiple epiphyseal dysplasia, thalassemia-related hemolytic anemia, and lysosomal storage disease. The diffused deformities in the upper-limb bones and several coarsened features of the craniofacial bones indicate the most likely diagnosis to have been a certain type of lysosomal storage disease. The skeletal remains of EP-III-4-No.107 from the Eunpyeong site, although incomplete and fragmented, provide important clues to the paleopathological diagnosis of skeletal dysplasias.

## Introduction

Skeletal dysplasia refers to a group of disorders that are characterized by abnormalities in the development, growth and maintenance of both bone and cartilage. These disorders are classified on the basis of the skeletal areas involved. Predominantly these include epiphyseal involvement (e.g., chondrodysplasia punctate, acromesomelic dwarfism, multiple epiphyseal dysplasia), metaphyseal involvement (e.g., achondroplasia, lysosomal storage diseases), and spinal involvement (e.g., spondyloepiphyseal dysplasia) [1]. Skeletal dysplasias related to genetic etiology have rarely been reported for past populations. Moreover, precise diagnosis of their causes is often impossible in archaeological cases. In paleopathology, accurate diagnosis depends greatly on the preservation and completeness of skeletal remains recovered from archaeological sites [1]. In this context, a detailed examination of skeletal dysplasia can provide valuable information for diagnosis of historical congenital dysplasias.

The aim of the current study is to analyze a case of skeletal dysplasia identified among a group of 198 individuals from a burial site in Eunpyeong, an area of Seoul, South Korea. The dysplastic individual was incomplete and fragmentary because the lower half of the rectangular burial pit was destroyed. Unfortunately, *in-situ* positions of the skeletal remains in the grave were unknown, as during the course of archaeological investigation, the skeletons had been disturbed. However, the individual showed clear signs of dwarfism-related skeletal dysplasia in the upper limbs. As far as we know, this is the first skeletal case of dwarfism in South Korea that has ever been examined. This report presents the skeletal features of an individual with dwarfism-related skeletal dysplasia in an effort to provide important clues to the diagnosis of congenital dysplasias in the paleopathological setting.

## Materials and Methods

**Fig 1:**
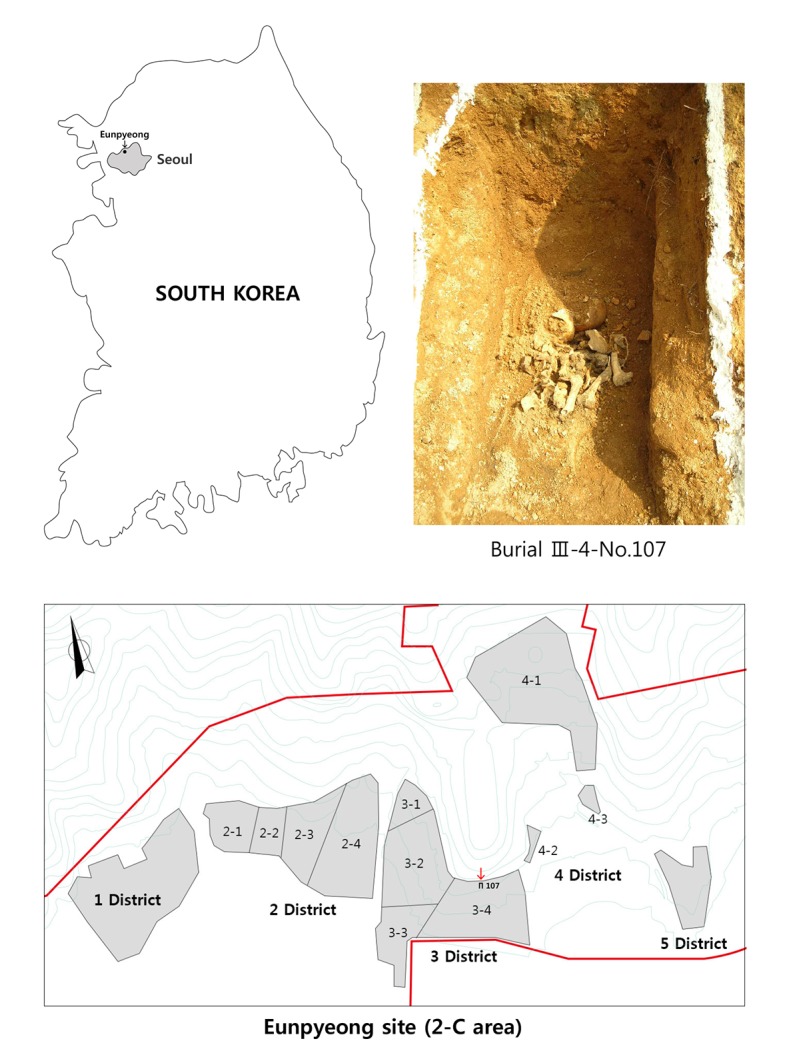
The location of the Eunpyeong site, in situ photograph of burial III-4-No.107 and the excavated districts (2-C area) Burial III-4-No.107 was 3–4 districted, is indicated by the red arrow.

The specimen (EP-III-4-No.107) under study was discovered in the III-4 district of the Eunpyeong cemetery (2-C area), which is located in the northeastern area of metropolitan Seoul, South Korea (Fig 1). The site was divided into five main districts. Archaeological investigation of this site was conducted from 2006 to 2007 by the Central Institute of Cultural Heritage and the Bio-anthropology Laboratory of Seoul National University (under the auspices of the Cultural Heritage Protection and Investigation Act of South Korea), in preparation of construction of a new town in Eunpyeong gu. Prior to the excavation, a public announcement was made based on the Funeral Services Related Act (Section 2) in order to find possible descendants of the individuals found at the site. As no skeletal remains had been claimed 60 days after the announcement, the excavation was allowed to proceed, and with permission of the Central Institute of Cultural Heritage, the skeletal remains were moved to the Bio-anthropology Laboratory of Seoul National University for detailed examination.

The burials were most concentrated in District III, which includes many multiple-burial sites. The 2-C area excavation recovered approximately 660 individuals. Among the individuals, 198 individuals are well-preserved. The Eunpyeong cemetery is a naturally constructed mortuary in the northeastern region of Seoul, due to a Joseon dynasty (AD 1392–1910) law that prohibited interment of the dead inside the capital. Analyses of burial types and artifacts suggest that this site was utilized for a relatively long period of time, from the mid-15th to the early 20th centuries [2]. Radiocarbon dating of a rib of EP-III-4-No.107 by Accelerator Mass Spectroscopy, undertaken by the National Center for Inter-University Research Facilities at Seoul National University, yielded a calibrated date range of 1780–1920 AD.

The individual under investigation in this study was discovered in a rectangular pit, approximately half of which had been destroyed due to the site’s use as a settlement since the early 1900s. In fact, the lower half of the burial had totally disappeared, along with the skeletal remains of the lower portion of the body. Also, EP-III-4-No.107 slightly overlapped with burial, EP-III-4-No.106. Given that the breadth of the burial pit was within the range of the general size of the wooden coffins at the Eunpyeong site, the individual was presumed to have been buried in a wooden coffin. The space between the wooden coffin and the wall of the pit was filled with a mixture consisting of a large amount of soil with a small amount of lime. The burial types within the Eunpyeong cemetery were of either the earth-pit (91%) or lime-mortar type (less than 10%). The individuals associated with the lime-mortar type of burial were better preserved than those in earth-pits, due to the air-tight environment created by the lime-soil mixture. Interestingly, lime-mortar burials were not found in the lower sections of the mountains. It is likely that use of this burial system had been restricted for individuals of a higher social status than the majority who were typically interred in simple earth-pits; lime at the time was difficult to manufacture and expensive [3]. It must be noted, however, that the lime-mortar grave in the present case differed from the norm at the Eunpyeong site, as large amounts of soil had been mixed with only a small amount of lime to fill the space between the wooden coffin and the wall of the pit. In light of this, the grave in the present case can be interpreted in two different ways. First, it might indicate that the social status of the individual was somewhat lower than those buried in the general lime-mortar type. Second, and irrespective of social class, the burial may represent a modified type that appeared during the transition process away from the pure lime-mortar type. During the mid-point of the Joseon Dynasty’, the lime-mortar burials gradually disappeared as a cultural practice. No archaeological artifacts were evident inside the Burial EP-III-4-No.107.

Due to the lack of pelvic elements, sex estimation of the individual was carried out on the basis of a macroscopic assessment of the skull [4]. More specifically, the nuchal crest, the mastoid process, the supraorbital margin, the glabella, and the mental eminence were considered [4–5]. The age at death was estimated by examining the palatal and ectocranial suture closures [6–7], dental wear [8] and degenerative changes of the pulp cavity [9]. Dental wear of preserved teeth was assessed according to the eight criteria of Smith [8], while the degenerative change of the pulp cavity was examined by measuring the length ratio of mandibular canines and left first premolar on radiographs [9]. All of the measurements were taken twice with an interval of two weeks and then averaged. For the craniometric analysis, re-assembly of the skull fragments was necessary. The lower occipital regions, particularly, were deformed and impossible to restore to their original shape. Therefore, the cranium was reconstructed using three-dimensional virtual software. Each cranial fragment was scanned by computed tomography (CT) (Dentri^®^ cone-beam CT, Willmed^®^, Republic of Korea) with a slice thickness of 1.0 mm and at increments of 0.5 mm. The skeletal images, formatted as Digital Imaging and Communications in Medicine (DICOM) data, were converted to stereolithography (STL) images using 3D visualization analysis software (Amira ^TM^ version 5.2.2 from VSG, USA). The image files were imported into a computerized 3D modeling system (Freeform Modeling^TM^ System software, 3D Systems Inc., USA) for virtual reconstruction. The reconstructed cranium was visually examined and craniometric measurements were taken. The cranial measurements and the cephalic index (CI) of EP-III-4-No.107 were compared to 62 skulls of normal female adults from the Eunpyeong site. The cranial thicknesses at the frontal, bregma, parietal and lambda regions were compared with Japanese data. Radiographs of the mandible and postcranial bones were taken at the Yonsei University College of Dentistry in Seoul. In order to examine the condition of the cortical bone and the morphology of the teeth in the relatively well-preserved mandible, dental panoramic tomography (Cranex 3+ Ceph, Soredex, Finland) was employed. Also, cone-beam CT scans (Alphard 3030, Alphard Roentgen, Japan) were taken of the completely preserved clavicles and humeri to examine the cross-sectional morphology. Scans of the clavicles were taken at the diaphyseal midshafts (50%) and the humeri at the diaphyseal midshafts and deltoideus muscle attachment site. The diaphyseal midshafts were determined from the maximum lengths of the bones. All of the cone-beam CT volumetric images were stored using DICOM 3.0 and subsequently transferred into the OnDemand 3D^TM^ application (Cybermed, USA). Axial volumetric slicing as well as 3D image reconstruction were used to select and explore representative images.

## Results

### Preserved skeletal elements and age and sex determination

**Fig 2:**
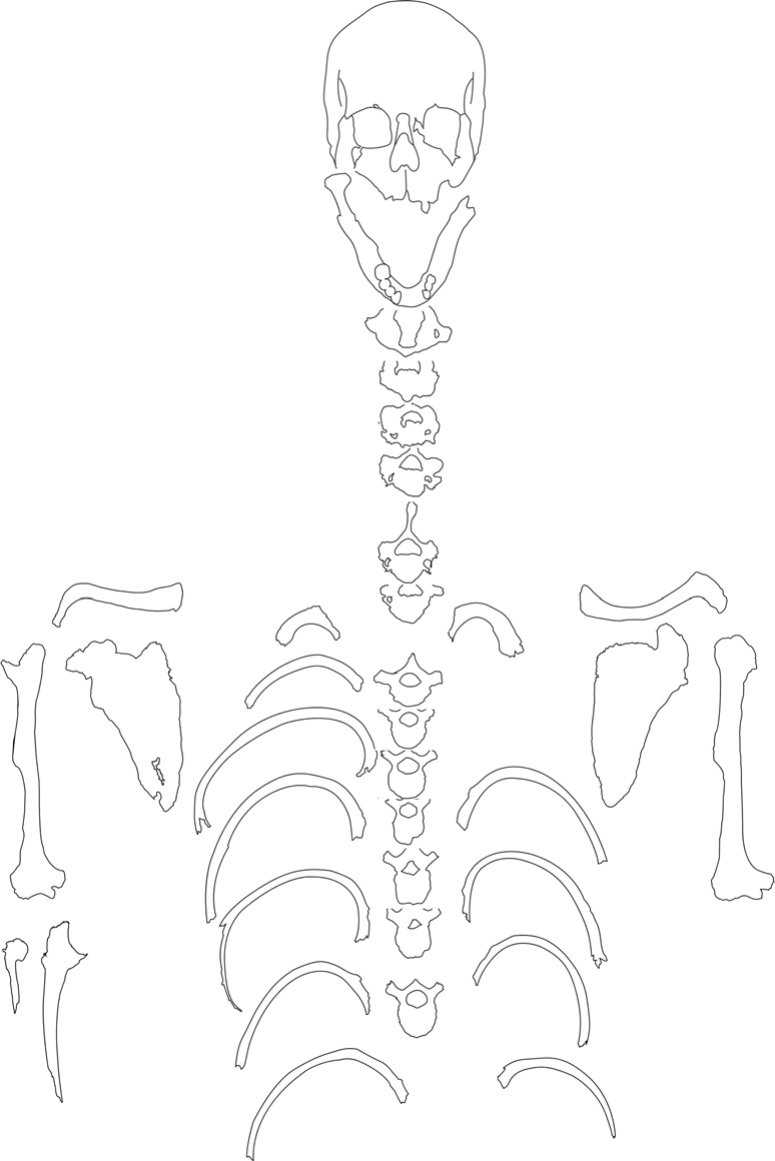
Preserved elements of III-4-No.107.

The individual is represented by the cranium, mandible, left and right clavicles, left and right scapulae, vertebrae (C1-4, 6–7, T2-7, 9), some ribs, left and right humeri, the right ulna shaft, and the right proximal radius (Fig 2). Overall, the cranium is nearly complete, missing only a portion of the left frontal and temporal bones, both the zygomatic arch, and a small part of the occipital bone posterior to the foramen magnum. The mandible is also mostly intact, except for the left condylar process and gonion. The postcranial remains are incomplete, limited exclusively to several upper body elements.

Although it was difficult to assess the sex due to the abnormal skeletal morphology and missing skeletal elements, several features suggest that this individual was very likely a female. The supraorbital tori are small and non-projecting, the forehead is vertical, and the mastoid process is relatively small and round. The mandible is small, and lacks a muscular ramus. The age at death was estimated to be approximately 36–50 years based on the ectocranial and maxillary suture fusion [6–7], degree of dental wear [8] and degenerative changes of the pulp cavity [9].

### Morphological and pathological analyses

**Fig 3:**
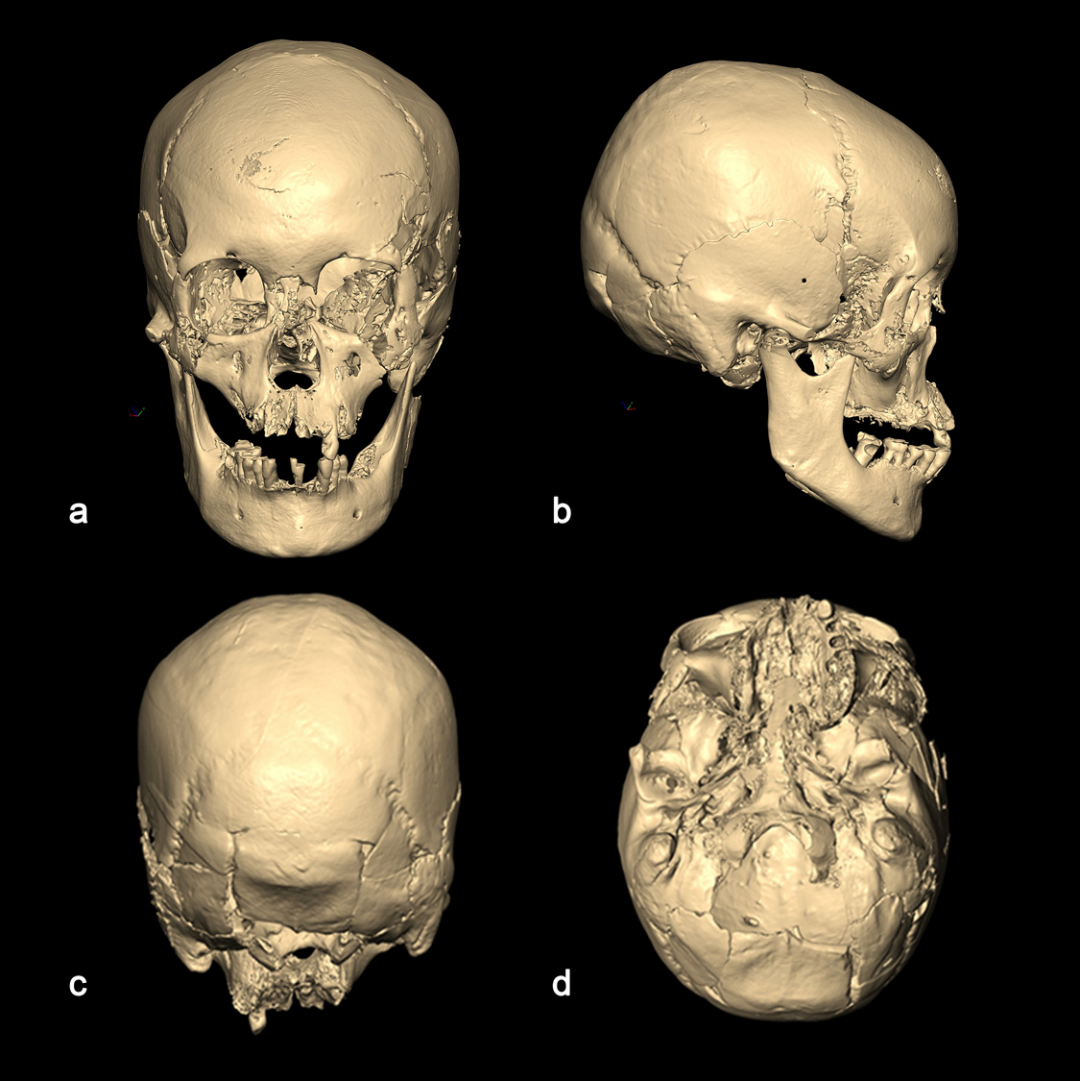
Reconstructed CT images of cranium (a, anterior view; b, lateral view; c, posterior view; d, basal view).

**Fig 4:**
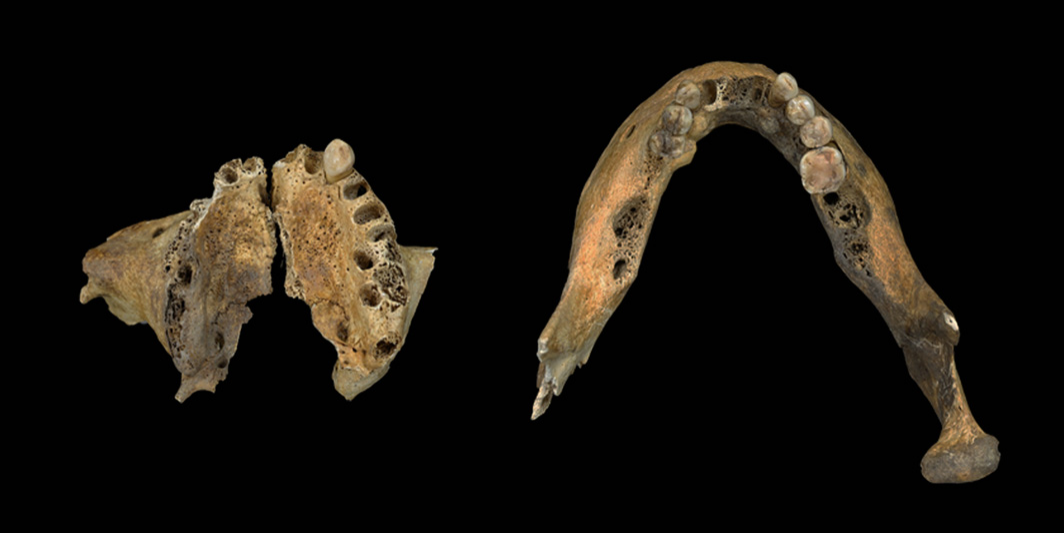
Maxilla and mandible.

The cranial vault shows marked frontal and parietal bossing, although the bosses are not greatly developed. From the anterior view, the frontal bone is relatively high and the upper face is flat. From the lateral viewpoint, the face is concave with a minor nasal projection, and pronounced alveolar prognathism (Fig 3). The mental eminence is receding rather than well developed. The general appearance of the mandible is normal, but the dental arcade is relatively narrow and the right mandibular condyle is more concave than the norm (Fig 4).

**Fig 5:**
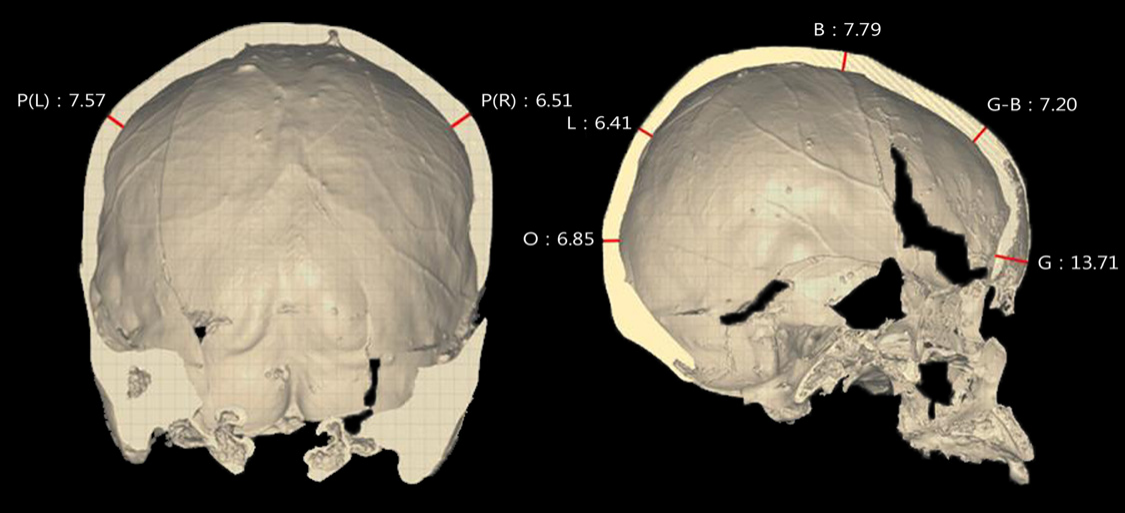
Sectional images of the cranial bone (Left, coronal section; Right, sagittal section) P(L), parietal (L); P(R), parietal (R); O, opistocranion; B, bregma; G-B, glabella-bregma (trichion); G, glabella.

Craniometric data are provided in Table 1. In general, most of the cranial dimensions for EP-III-4-No.107 are larger than other female crania from the site, though with some exceptions (e. g., biauricular breadth, nasal height and breadth, parietal chord, and right mastoid length). Specifically, the maximum cranial length and the right orbital breadth are outside of the normal female range. The cranial index of this individual indicates a mesocranic type (CI = 78.8), whereas normal Eunpyeong females are brachycranic (CI = 81.8, N = 62, 136.6/167×100). All of the mandibular dimensions are larger than those of normal females. Additionally, all the cranial vault elements are unusually thick: the thickest is found on the glabella region (13.71mm) of the sagittal section plane (Fig 5). Finally, slight porotic hyperostosis on the ectocranial parietal and occipital surfaces and cribra orbitalia on the orbital roof was noted (S1 Fig).

**Table 1:** Metric data for EP-III-4-No.107 compared with normal females from the Eunpyeong site.

	EP-III-4-No.107		Normal females (N = 62)	
*Craniometric data* (mm)	Measurements	% of compared sample mean	Mean	Range
Maximum cranial length	176.9	105.9	167.0	147–176
Maximum cranial breadth	139.5	102.1	136.6	126–151
Cranial base length	99.9	106.7	93.6	85–100
Basion-prosthion	99.3	108.6	91.4	80–106
Biauricular breadth	101.0	97.2	103.9	94.5–112
Minimum frontal breadth	92.9	105.0	88.5	78.0–97.8
Upper facial breadth	102.2	103.6	98.6	93.6–106.4
Nasal height	48.4	98.8	49.0	42.1–54.6
Nasal breadth	22.4	89.6	25.0	22.4–28.1
Orbital height	L: 35.6R: 35.3	L: 105.0R: 103.2	L: 33.9R: 34.2	L: 29.9–37.8R: 30.4–38.4
Orbital breadth	R: 40.0	R: 106.7	R: 37.5	R: 34.0–39.3
Biorbital breadth	93.8	108.1	86.8	81.0–98.1
Frontal chord	109.2	102.5	106.5	96.4–113.3
Parietal chord	106.5	99.0	107.6	95.7–122.5
Mastoid length	L: 26.5R: 24.4	L: 100.8R: 93.1	L: 26.3R: 26.2	L: 20.6–35.6R: 22.2–31.6
Chin height	35.8	117.8	30.4	26.3–36.9
Body height at mental foramen	L: 33.5R: 33.3	L: 115.5R: 112.9	L: 29R: 29.5	L: 24.4–36.2R: 24.9–35.7
Body thickness at mental foramen	L: 13.8R: 14.6	L: 120R: 125.9	L: 11.5R: 11.6	L: 8.9–14.0R: 8.9–15.0
Minimum ramus breadth	R: 33.0	R: 104.4	R: 31.6	R: 27.2–35
Maximum ramus breadth	R: 44.2	R: 105.5	R: 41.9	R: 35.7–47.1
Maximum ramus height	R: 62.7	R: 117.8	R: 53.2	R: 41.2–68.2
Mandibular length	74	104.4	70.9	60–77.5
*Post-cranial metric data* (mm)				
Clavicle				
Maximum length	L: 134R: 118	L: 101.9R: 91.8	L: 131.5R: 128.5	L: 114–142R: 112–135
Sagittal diameter at midshaft	L: 9.1R: 9.4	L: 82.7R: 88.7	L: 11.0R: 10.6	L: 9.1–12.7R: 8.2–13.0
Vertical diameter at midshaft	L: 9.5R: 7.4	L: 104.4R: 86	L: 9.1R: 8.6	L: 7.2–10.5R: 7.6–9.4
Humerus				
Maximum length	L: 226R: 209	L: 81.8R: 75.7	L: 276.4R: 275.9	L: 230–304R: 228–312
Maximum diameter at midshaft	L: 21.1R: 23.1	L: 111.6R: 120.3	L:18.9R: 19.2	L: 16.3–22.8R: 16.4–22.8
Minimum diameter at midshaft	L: 15.8R: 13.8	L: 108.2R: 95.2	L: 14.6R: 14.5	L: 12.3–18.8R: 12.1–17.4

**Fig 6:**
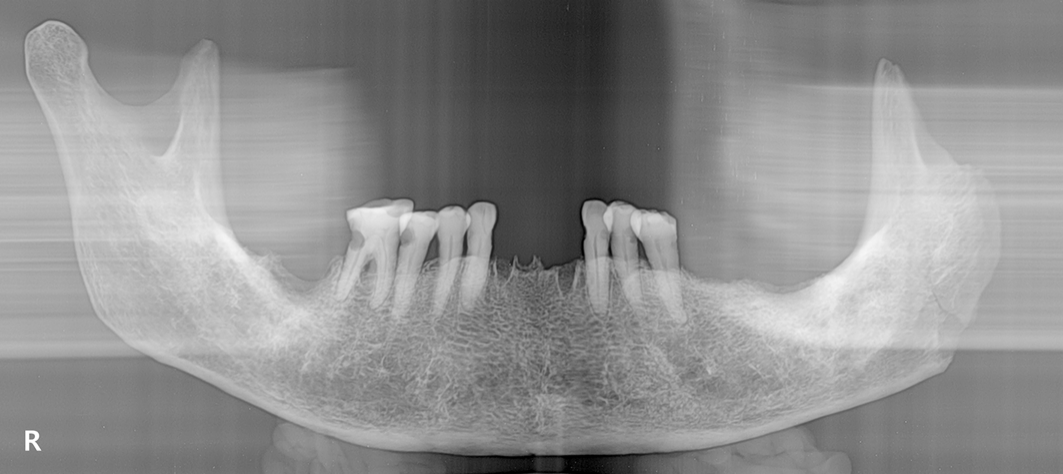
Panoramic radiographs of mandible.

In the maxilla, only the left canine is present, with carious lesions. Abscesses are evident on the alveolar bone of the right premolars, the left first premolar, and the left first and second molars. Also, there is ante-mortem loss of the right first and second premolars, right first and second molars, and left first and second molars. In the mandible, ante-mortem tooth loss, linear enamel hypoplasia, caries and abscesses are observed (S2 Fig). Both of the central incisors and the left first, second and third molars as well as the right second and third molars had been lost ante-mortem. Multiple linear hypoplastic defects are observed on the left and right canines and the left first and second premolars. Carious lesions were present on the right second premolar and first molar, and the left canine and premolars. Finally, abscesses were noted on the alveolar bone of the right second molar, and mandibular tori were found on both sides of the mandibular body. Dental panoramic tomography images showed no evidence of any abnormal condition of the cortical bone or dental morphology (Fig 6).

**Fig 7:**
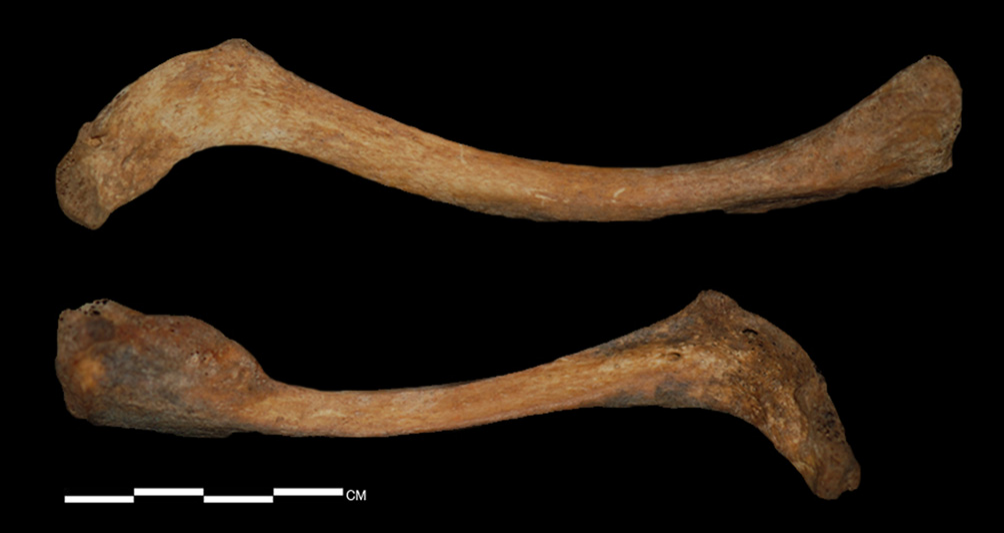
Superior surface of clavicle (upper, left side; lower, right side).

**Fig 8:**
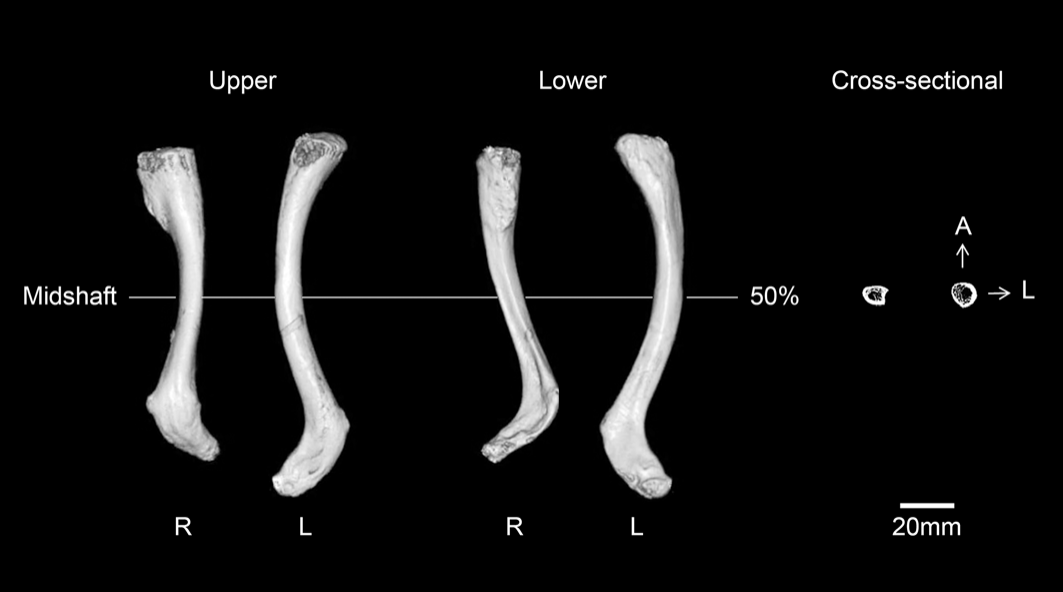
Reconstructed CT images and cross-sectional images of the clavicles (A, anterior; L, lateral).

**Fig 9:**
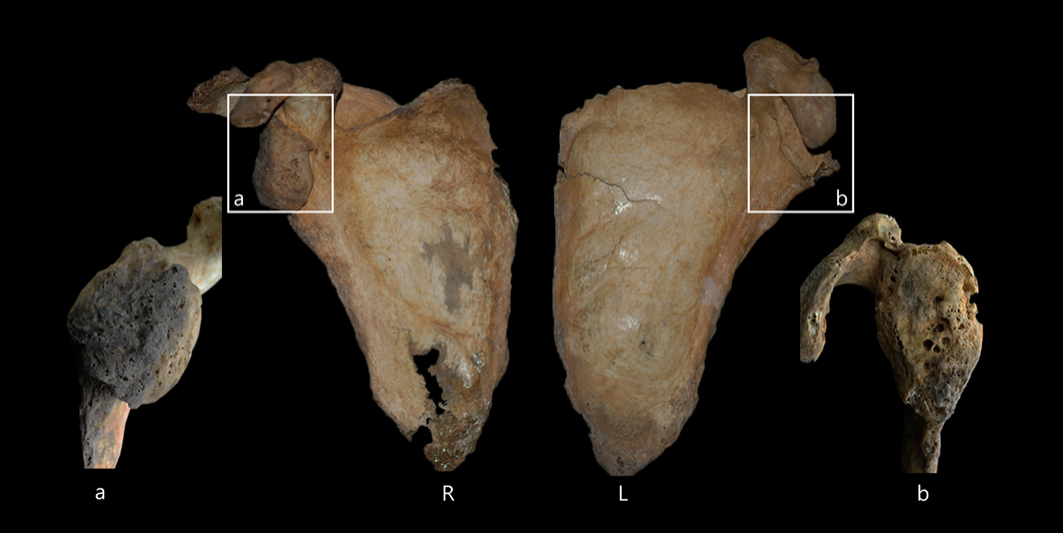
Anterior view of scapula (a, right glenoid cavity; b, left glenoid cavity).

The two sides of the clavicles display differences in form, which are particularly evident on the medial and lateral ends (Fig 7). The postero-inferior midshaft of the right clavicle, unlike the left, is flattened and angulated with a clear subclavian groove. Both clavicles are uncommonly wide in the medial portions, but this is more pronounced in the right clavicle. The maximum length is longer in the left than in the right (Table 1). Also, at the attachment site of the deltoideus muscle of the left clavicle, abnormal foramen and periosteal reactions surrounding the foramen were observed. CT images of the clavicles demonstrate evident asymmetry in the diaphyseal midshafts as well as asymmetry in the clavicular length (Fig 8). The left midshaft has a more round shape in the cross-sectional morphology. Both scapulae exhibit severe malformation in the glenohumeral joint, with an irregular and pitted subchondral plate (Fig 9). Additionally, both coracoid processes show abnormal porosity and perforation. The individual probably had a functional disorder of the shoulder joints.

**Fig 10:**
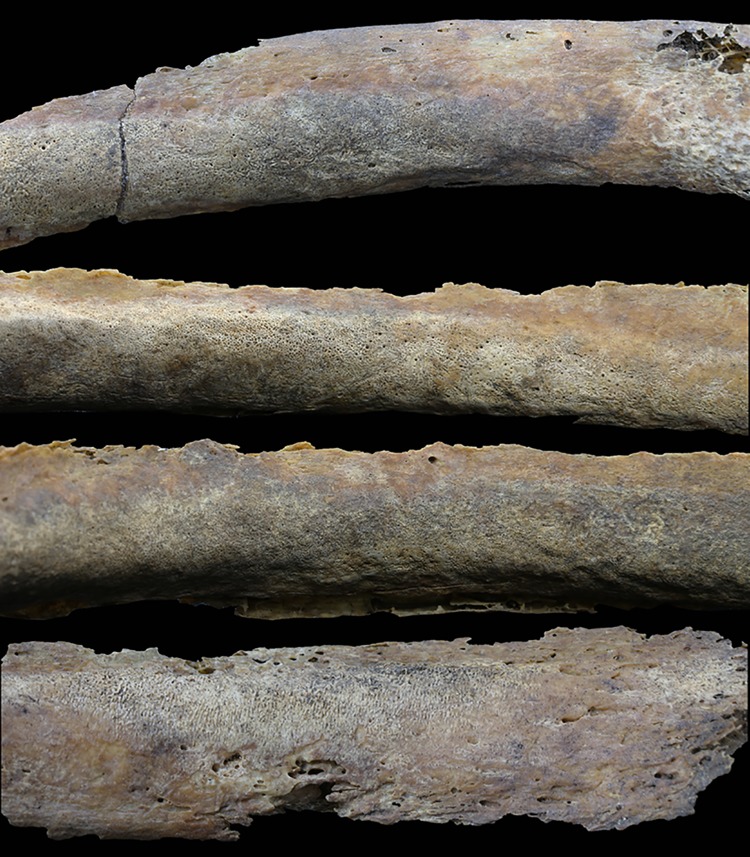
Right ribs showing periosteal reactions along their shaft.

The cervical and upper thoracic vertebrae demonstrate a generally normal morphology. The vertebral bodies are small, but their height is normal, and the size of the neural arches is normal. In the cervical vertebrae, degenerative changes in the apophyseal and intervertebral joints were clearly observed. In particular, the right apophyseal joints of the third and fourth cervical vertebrae display severe osteophytosis and moderate porosity. In the thoracic vertebrae, the vertebral body endplates in the sixth, seventh and ninth thoracic vertebrae exhibit Schmorl’s nodes. Some ribs are present but are in a fragmented condition. The right ribs on the visceral surface show a rough texture and periosteal reactions along the shaft. Six ribs have proliferative lesions with a mixture of woven and lamella bone along the shaft (Fig 10).

**Fig 11:**
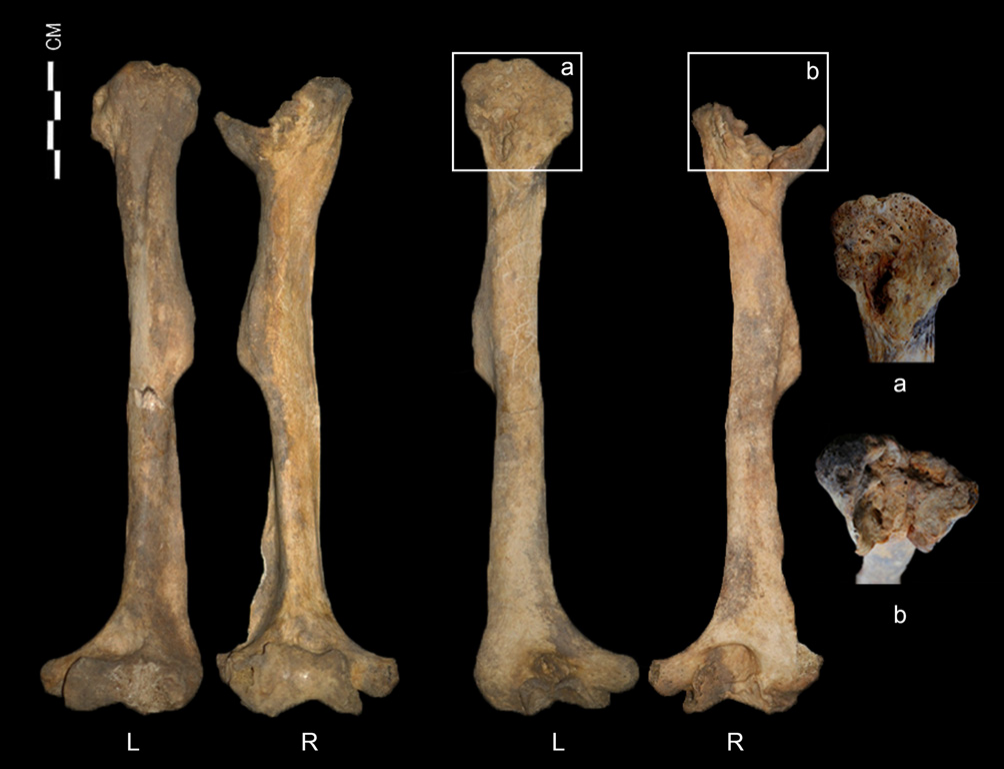
Anterior and posterior views of the humerus (a, left head from a posterior view; b, right head from a superior view).

**Fig 12:**
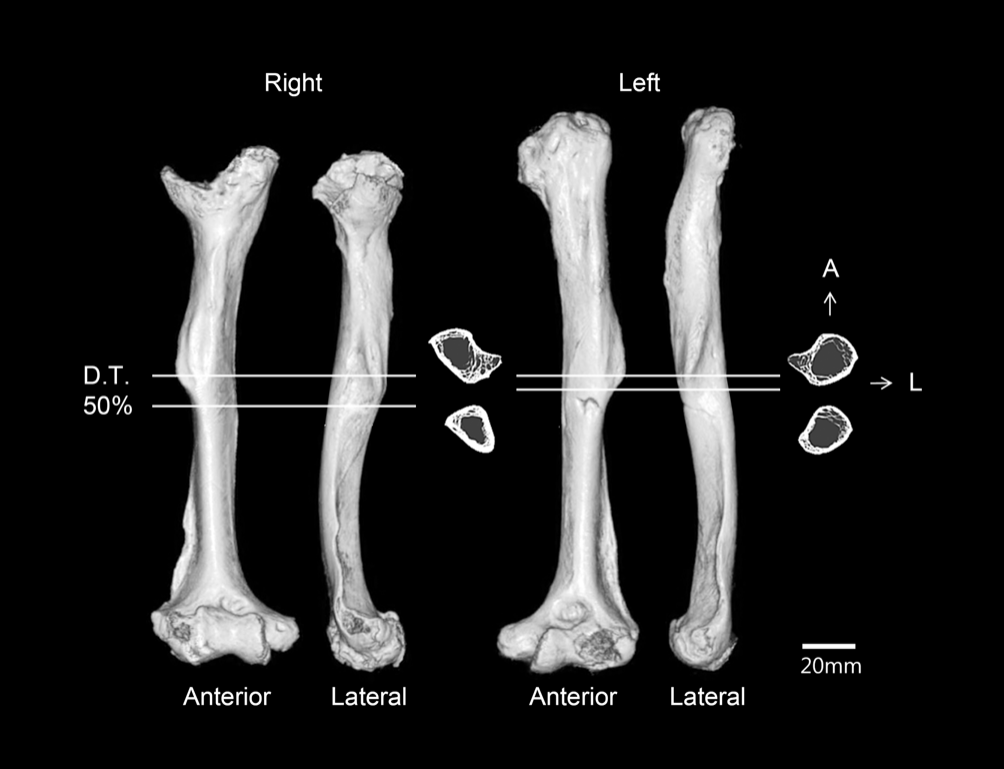
Reconstructed CT images and cross-sectional images of the bilateral humeri (D.T., deltoid tuberosity; A, anterior; L, lateral).

The humeri show the greatest deformity of all of the skeletal elements, with both the diaphysis and epiphysis affected (Fig 11). Both humeri are abnormally short with excessively angulated deltoid tuberosity. The right humerus is much shorter than the left (about 17 mm) (Table 1). The greatest difference is in the maximum length of the right humerus, which is only 76% of normal females from the same cemetery. The humeral heads exhibit severe malformation of the articular surface, with pitting of the subchondral plate. The two sides of the humeral heads are differently shaped. The head of the left humerus has a longitudinally flat medial surface, while the right head is scooped, with edges on the medial and lateral borders. The head of the left humerus is directed posteromedially such that the posterior surface of the diaphysis is displaced laterally. Given the morphology of both heads, it is clear that they had been dislocated from their original articulation. Also, asymmetric patterns are present in the olecranon fossae and lateral condyles of the humeri. The olecranon fossa of the left is shallower than that of the right, while the lateral condyle is more developed in the right than in the left. Osteoarthritic changes are severe in both elbow joints. Osteophytes are observed in the left distal articular surface, and osteophytes and eburnation are noted in the distal end of the right humerus. Finally, on CT scans, the midshafts and the deltoid tuberosities of the humeri are relatively thin. Specifically, the midshaft region of the left is thinner than of the right (Fig 12).

**Fig 13:**
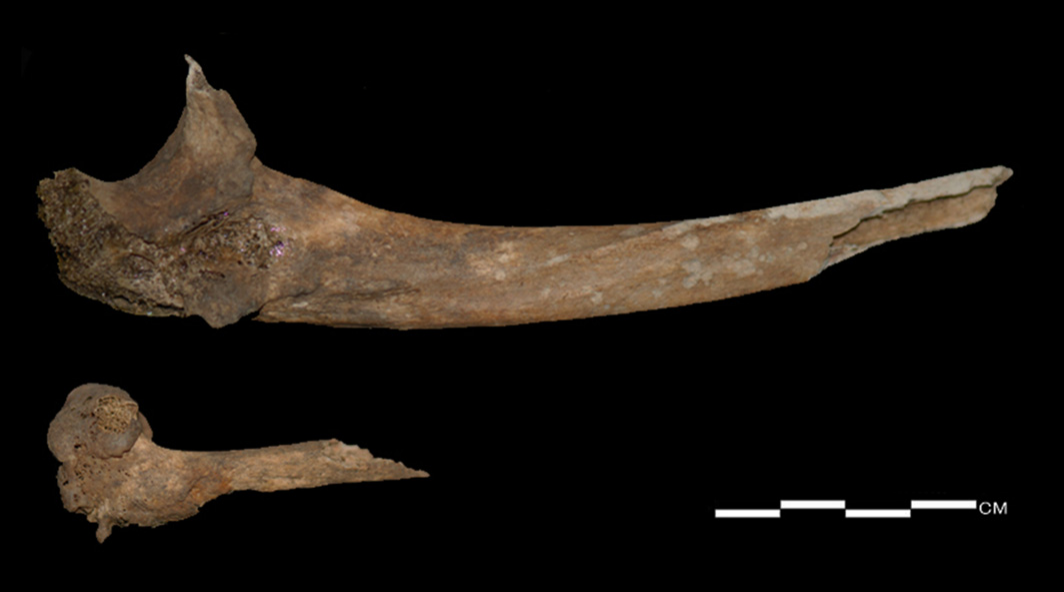
Right ulna and radius.

The radius and ulna also show deformities, more severe in the radius than in the ulna. The head of the radius is small and extremely deformed, with a very rugged shape (Fig 13). Considering the shape of the radial head, it appears that the radiohumeral and radioulnar joints had been dislocated. Also, the proximal shaft is abnormally slender. The ulna is characterized by a large trochlear notch and a small facet of the radial notch (Fig 13). There is a severe lytic lesion between the radial notch and the lateral margin of the coronoid process. Additionally, osteoarthritic changes such as porosity and eburnation are evident in the trochlear articular surface of the ulna. The proximal ulna shaft likely bows in the anterior direction, although it is unclear due to the missing distal end.

## Discussion

As skeletal dysplasias are a considerably heterogeneous group of genetic disorders (more than 200 different entities having been identified to date) affecting skeletal morphogenesis and development [10], its diagnosis can be difficult. In this study, we carefully examined specimen EP-III-4-No.107 through macroscopic and radiographic evaluation. The deformities of the humeri and glenohumeral joints are typical features of humerus varus deformity (HVD) [11–12]. Furthermore, the skeletal dysplasia in this case occurred in the epiphyseal plates of the clavicles, the scapulae, the radius and the ulna. The affected glenohumeral and radiohumeral joints were severely deformed, which probably had significant effects on the use of the shoulder and elbow joints during the individual’s lifetime. Also, the skeletal abnormalities in the craniofacial bones included cranial thickening, frontal and parietal bossing, and alveolar prognathism. However, there were no abnormalities in the vertebral columns or the ribs. We compiled a list of conditions that cause significant dysplasias in epiphyseal plates and metaphyses, with respect to the HVD in this case. Diagnoses were made solely on the skeletal manifestations, and conditions that were inconsistent with key aspects of probable pathologies were ruled out as possibilities. With regard to the shortened limbs and abnormal cranial features, such as bulging of the forehead and alveolar prognathism, achondroplasia or hypochondroplasia and chondrodysplasia were considered as plausible diagnoses. For severe deformities in the epiphyseal plates along with HVD, multiple epiphyseal dysplasia (MED), thalassemia-related hemolytic anemia and lysosomal storage disease (LSD) were considered as differential diagnoses.

### Achondroplasia or hypochondroplasia

Achondroplasia is the most common form of short-limb dwarfism, showing an incidence between one in 10,000 and one in 30,000 [13–14]. In the paleopathological record, achondroplasia has been relatively well-documented in a range of archaeological skeletons [15–20]. Achondroplastic individuals are characterized by a rhizomelic disproportionately short stature, an enlarged head, an undersized skull base, midface hypoplasia, a depressed nasal bridge, short hands and lumbar lordosis [1, 14, 21–22]. Hypochondroplasia, meanwhile, is a milder form of short-limb dwarfism. This condition usually is differentiated from achondroplasia by the lack of changes to the facial skeleton [20–21].

Some craniofacial features in the present case are consistent with a diagnosis of achondroplasia. More specifically, the prominent forehead, concavity of the facial region and the protruding mandible of EP-III-4-No.107 are typical features of disproportionate dwarfism such as achondroplasia. Also, the alveolar prognathism noted in this case is frequently observed in achondroplastic conditions [15]. However, the skull base of EP-III-4-No.107, including the area surrounding the foramen magnum, clearly was not constricted or depressed, as noted in most cases identified as achondroplasia [19–20]. Additionally, the occipital condyles were not flat, and the size of the foramen magnum was not abnormally small, as seen in conditions of disproportionate dwarfism such as achondroplasia or hypochondroplasia.

In Burial EP-III-4-No.107, the maximum length of the humeri was shortened, ranging from 18.2% (left) to 24.3% (right) shorter than the normal mean size of females from the same site. However, in the rhizomelic pattern, the proximal limb bones are short, ranging from 20% to approximately 60% of the normal size [15, 20]. Given this, the shortening of the humeri of EP-III-4-No.107 appears minimal. Additionally, wide epiphyses and metaphyses of the distal portion of the humeri should be considered a skeletal manifestation of achondroplasia or hypochondroplasia on differential diagnoses. Burial EP-III-4-No.107 does not display these features.

The most obvious inconsistency between achondroplasia or hypochondroplasia and the case examined here was in the contours of the joint surfaces. Patients with achondroplasia typically show normal contours of the humeral heads, though varus inclination commonly occurs [11, 23]. Achondroplastic or hypochondroplastic conditions merely show a flattened form of the proximal articular heads of the long bones, rather than gross deformations of the proximal epiphyses. Hence, the normal aspects of EP-III-4-No.107’s cranial base and the severely deformed contours of the proximal epiphyses in the humeri and radius rule out achondroplasia or hypochondroplasia.

### Chondrodystrophic dwarfism

In general, this type of short-limb dwarfism differs from achondroplasia in that the greatest length-reduction of limb bones occurs in the ulna and radius, even though their cranial abnormalities are similar [24]. All chondrodystrophic individuals share the common characteristic of a reduced stature, but clinicians classify types according to whether all of the limbs are short or if the proximal, middle, or distal limb segments are disproportionately affected [24–25]. In the paleopathological record, a well-known case of chondrodystrophic dwarfism is that of a female (Romito 2) from an upper Paleolithic site in Italy [24]. In complete correspondence with the conditions of achondroplasia or hypochondroplasia, that specimen showed brachycephaly, anterior bulging of the forehead, and a reduction in the cranial base. The humeral head was elliptically shaped, and the radius and ulna were the most shortened of all of the postcranial remains, unlike cases of achondroplasia or hypochondroplasia. Clinical cases of chondrodystrophic dwarfism also manifest acromesomelic or mesomelic shortening in the limb bones, with some extremities showing marked hypoplasia. However, the upper-limb bones in clinical cases are not abnormally shaped, as seen in the present case, though they are somewhat poorly developed in their proportions [26–29].

Individual EP-III-4-No.107 clearly showed deformity of the radius and ulna. However, the greatest deformity was present in the humeri, but due to the missing distal parts in the radius and ulna, it was impossible to determine which upper-limb segment was the most shortened. Along with the difference in the limb bone deformation pattern, our case did not show any of the skull-based depressions typically seen in chondrodysplasia. For these reasons, chondrodysplasia also was ruled out.

### Multiple epiphyseal dysplasia

MED significantly affects the epiphyses of tubular bones including the metacarpals, metatarsals and phalanges, whereas the metaphyses and vertebral bodies are impacted only slightly or not at all [30]. Additionally, MED patients are clinically characterized as having hip dysplasia and thoracic kyphosis, but normal cranial and facial morphology. Sometimes, spinal involvement such as scoliosis occurs, but in MED, this is restricted to the thoraco-lumbar region [1, 21–23, 31–33]. In MED, the irregular epiphyseal growth affects many joints, forming flattened and dysplastic articular surfaces and finally leading to early-onset osteoarthritis [34–35]. Clinical data report that the affliction is symmetrical, commonly involving the hips, knees, shoulders and ankles [36]. This symmetry has been reported in a young adult male from an Old Kingdom cemetery in Egypt [1].

The humeri and clavicles of EP-III-4-No.107 showed asymmetrical shortening and skeletal dysplasia. Particularly, in the humerus, the left head was longitudinally flat, while the right was concave with edges on the medial and lateral borders. The radius of EP-III-4-No.107 was severely deformed in the proximal portion, and the ulna showed abnormal articular surfaces. Moreover, there were no vertebral abnormalities, nor was there evident scoliosis, although all skeletal elements below the ninth thoracic vertebral column were missing.

### Thalassemia-related hemolytic anemia

In this study, the abnormal morphology of the humerus of individual EP-III-4-No.107 was closely aligned with typical clinical cases of proximal humerus varus, as it displayed nearly complete failure in epiphyseal development. HVD can be an important clue to the diagnosis of ancient diseases, in spite of being associated with a wide range of etiologies [12, 23, 37]. HVD occurs in patients with thalassemia-related hemolytic anemia, which varies in prevalence from 14% to approximately 50% [11, 23, 38]. Clinical studies suggest that premature fusion of the growth plates in the limb bones is a common finding in children with thalassemia [39]. Varus proximal head deformity, including HVD, is regarded as the result of extruded subperiosteal marrow in the area where the metaphysis joins the epiphysis to obliterate the medial portion of the physeal plate [38]. A case of possible congenital hemolytic anemia with glenohumeral joint involvement similar to EP-III-4-No.107 has been reported in a young individual from prehistoric Israel [40]. The flat and irregular humeral head and rugged glenoid surfaces of the scapula resembled the humeri and glenohumeral joints in EP-III-4-No.107, except that the right and left aspects of the humeri in the present study were very different.

The most obvious inconsistency between thalassemia-related hemolytic anemia and the present case is the absence of lytic cortical defects (i.e., extensive pitting) in the shafts of Burial EP-III-4-No.107’s long bones. Congenital hemolytic anemia such as thalassemia, produces lytic cortical defects including extensive porosity of the shaft of most long bones [41]. These cortical defects are presumed to result from the spread of hyperplastic marrow through the cortex of immature bone [40]. EP-III-4-No.107 did not display these lesions. Also, thalassemia causes marked thickening of the cranial vault, with the resultant “hair-on-end” appearance, porotic hyperostosis, and enlargement of the marrow space in the long bones [42–43]. According to genetic studies, the most common form of thalassemia (ß-thalassemia) mainly occurs predominantly in people from the central and eastern Mediterranean regions [12, 40], even if global migration has led to its spread around the world [42]. Currently, in South Korea, the carrier frequency of ß-thalassemia is reported to be only around 0.1% [44]; furthermore, the Korean climate is unsuitable for the maintenance of thalassemia. Hence, the deformities of the humeri and glenohumeral joints in EP-III-4-No.107 are unlikely to have been caused by thalassemia.

### Lysosomal storage disease

Another diagnosis to be considered is LSD. LSDs are a group of inherited metabolic disorders characterized by impairment of the intralysosomal catabolic pathways [45]. LSDs are multi-systemic diseases, and a major morbidity factor can develop as a consequence of involvement of the cardiopulmonary and nervous systems and skeleton [46]. Although there are more than 50 individual LSDs, historically, their characteristic skeletal abnormalities have been described in three broad categories: mucopolysaccharidoses (MPS), mucolipidoses, and oligosaccharidoses [47]. MPS and mucolipidoses occur in a variety of clinical manifestations with overlapping abnormal skeletal features, and can be indistinguishable in archaeological skeletons [42]. The prevalence of LSDs is 1 in 7,100–7,700 live births, of which MPS accounts for around 35% [48]. Other LSDs (e.g., especially oligosaccharidoses, glycoprotein degradation disorders) and carbohydrate-deficient glycoprotein disorders cause abnormal joint and skeletal development [49]. The clinical manifestations of LSDs vary, but often include typically progressive skeletal abnormalities [48]. Among the various subtypes of LSDs, most MPSs and mucolipidoses exhibit the type of bone malformations collectively known as dysostosis multiplex [48]. Dysostosis multiplex involves bones with abnormal shapes in nearly all skeletal elements [50]. Almost all MPS patients exhibit skeletal abnormalities, which include an enlarged skull, hypoplastic epiphyses, a thickened diaphysis, thoraco-lumbar kyphosis and hip dysplasia [50]. In particular, thoraco-lumbar kyphosis or the gibbus deformity caused by hypoplastic vertebral bodies is a key diagnostic feature [51].

In our study, skeletal abnormalities of the clavicles, scapulae, humeri, radius and ulna were suggestive of dysostosis multiplex. Dysostosis multiplex, characterized specifically by bone thickening and abnormal ossification of the epiphyses in multiple anatomic regions, is clinically diagnosed by radiological manifestations such as destructive bone and cartilage erosions, joint space narrowing and soft tissue swelling [47, 52]. In relation to these skeletal manifestations, joint stiffness and contractures can be found in most cases of LSD, and are most likely caused by metaphyseal deformities [52]. Increased chondrocyte apoptosis leads to abnormal articular cartilage matrix and subsequent progressive degenerative joint disease [52].

Overall, there are important similarities between the skeletal manifestations of MPS and the skeletal features in this case. Specifically, the destructive metaphyseal deformities in the glenohumeral joint and the severe degenerative changes in the elbow joint of EP-III-4-No.107 are suggestive of MPS’s skeletal manifestations. It has been demonstrated that disturbance of endochondral bone formation is exacerbated at specific sites by abnormal biomechanical forces that can arise from joint deformities [52–53]. This condition can affect joint mobility. In the individual in the present study, the right glenohumeral joint was more severely dislocated from its original articulation, with a more severe limitation of glenohumeral joint abduction than the left side. As the deformity worsens, the abduction and forward flexion of the glenohumeral joint become limited by the impingement of the greater tuberosity against the acromion [54]. Presumably due to abnormal biomechanical stress in the shoulder, EP-III-4-No.107 also manifested severe osteoarthritic changes in the elbow joint.

The thickened cranium, as observed in EP-III-4-No.107, is commonly reported in MPS patients [32, 52, 55–58]. The cranial thickness in this individual was compared with data from a Japanese population (data from Korean populations do not exist). Specimen EP-III-4-No.107’s cranium was much thicker than those of modern Japanese and the Jomon population in the frontal and parietal regions (Table 2). Furthermore, the craniofacial features included a prominent forehead, a slightly flattened nasal bridge and alveolar prognathism. Nearly all MPS patients show some degree of coarsening of facial features (e.g., a large head with a prominent forehead, round eyebrows, a flattened nasal bridge, widely spaced teeth, and prognathism) [60]. However, it is unfortunate that in this case, the lower body including the lower thoracic region and all of the lumbar vertebrae were not recovered, making it impossible to determine if this individual had a gibbus deformity, characteristic of MPS.

**Table 2:** Comparisons of cranial thickness between EP-III-4-No.107 and Japanese.

Population	Point			
	Frontal*	Bregma	Parietal**	Lambda
EP-III-4-No.107	7.2	7.8	7.6	6.4
Jomon Japanese female (N = 18) [59]	5.6	7.9	6.2	8.0
Modern Japanese female (N = 47) [59]	4.9	6.0	5.7	7.2

Frontal*, the midpoint of the distance between glabella and bregma on the frontal region

Parietal**, the most lateral points on the both parietal curvatures created by the coronal plane intersecting the vertex (left)

The internal surfaces of some of the ribs of EP-III-4-No.107 displayed proliferative lesions with a mixture of woven and lamellar bone. These lesions resembled reported cases of tuberculosis (TB) and other chest infections [61–62]. Although direct evidence has not been found, the immunity deficit caused by LSD might make patients more susceptible to mycobacterial infections [63]. Relatedly, MPS VІ complications include recurring episodes of pneumonia with features of obstructive and restrictive lung disease [57] and Hurler-Scheie syndrome, which is an intermediate form of MPS that causes serious abnormalities of the upper airways and lung [64]. Moreover, respiratory failure is the primary cause of death in nearly two-thirds (63%) of patients with Morquio syndrome A (MPS VІ A), [65]. In this context, it is possible that this individual died due to a complication related to pulmonary disease.

A comparative analysis of the distribution of the skeletal abnormalities exhibited in this case (Table 3) leads to a most likely diagnosis of LSD. In LSD patients, the most important symptoms occur in the skeletal system with multiplex dysostosis. Usually, the long bones are characterized by several alterations: the diaphyses are shortened and curved in the distal part; the epiphyses, meanwhile, are hypoplastic and thinned cortically by osteoporosis [66]. Some of the cranial features of EP-III-4-No.107, as well as the shortened humerus, resemble disproportionate dwarfism. Furthermore, multiple malformations predominantly in the metaphyses and epiphyseal plates are suggestive of MED and thalassemia-related hemolytic anemia. However, striking differences exist between EP-III-4-No.107’s skeletal manifestations and those in disproportionate dwarfism, MED and thalassemia-related hemolytic anemia. In this individual, although not all of the features commonly found in LSDs were observed, the distribution and severity of skeletal deformities exhibited certainly are strong indications of LSD.

**Table 3:** Skeletal features associated with skeletal dysplasias and in EP-III-4-No.107.

Features	Achondro-plasia	Chondro-dysplasia	MED	Thalassemia	LSD	EP-III-4-No.107
***Skull***						
Macrocephaly	√			√	√	
Thickened cranial vault				√	√	√
Bulging forehead	√	√			√	√
Constricted cranial base	√	√				
Depressed nasal bridge	√	√			√	√
Protruding mandible	√	√			√	√
***Appendicular***						
Shortened limbs	√	√	√		√	√
Thick irregular clavicles					√	√
Humerus varus			√	√	√	√
Genu varum	√		√	√		N/A
Hypertrophies at muscular attachment	√	√	√		√	√
Lytic cortical defect in the shaft				√		
Secondary osteoarthritis			√		√	
***Axial***						
Odontoid hypoplasia					√	
Short vertebral bodies		√				
Thoraco-lumbar scoliosis			√			N/A
Thoraco-lumbar kyphosis	√				√	N/A
Shortened trunk						N/A

√ means osteological features are present

N/A = not applicable

## Conclusions

The skeletal remains of EP-III-4-No.107 from the Eunpyeong site, albeit incomplete and fragmented, provide important clues to the paleopathological diagnosis of congenital dysplasias. We believe that morphological studies of skeletons can be helpful in further narrowing the differential diagnosis of skeletal dysplasias. In determining the etiology of the shortened limbs and abnormal cranial features of EP-III-4-No.107, we considered achondroplasia or hypochondroplasia and chondrodysplasia as possibilities. However, contraction of the skull base coupled with the normal contours of the joint surfaces, appearing with the above conditions, represented a serious inconsistency. In determining the etiology of the severe deformities of the epiphyseal plates along with humerus varus deformity (HVD), multiple epiphyseal dysplasia (MED), thalassemia-related hemolytic anemia and lysosomal storage disease (LSD) were considered as differential diagnoses. MED was rejected due to the asymmetrical shortening and skeletal dysplasia observed in this case. Finally, thalassemia-related hemolytic anemia was rejected, because there were decisively no traces of lytic cortical defects, such as extensive porosity, in the preserved long bones. Given the diffused deformities of the upper- limb bones and the coarsened facial features, we argue that a type of lysosomal storage disease is most consistent with the suite of traits observed here.

## Supporting Information

S1 Fig The left orbital roof of III-4-No.107. (TIF)

S2 Fig Mandible of III-4-No.107: multiple linear hypoplastic defects on right canines, carious lesions on the right second premolar and first molar. (TIF)
