# Supplementary figures and images for: Paleopathological Study of Dwarfism-Related Skeletal Dysplasia in a Late Joseon Dynasty (South Korean) Population
Source: PLoS One. 2015 Oct 21;10(10):e0140901. doi: 10.1371/journal.pone.0140901 (PMC4619213; doi:10.1371/journal.pone.0140901)

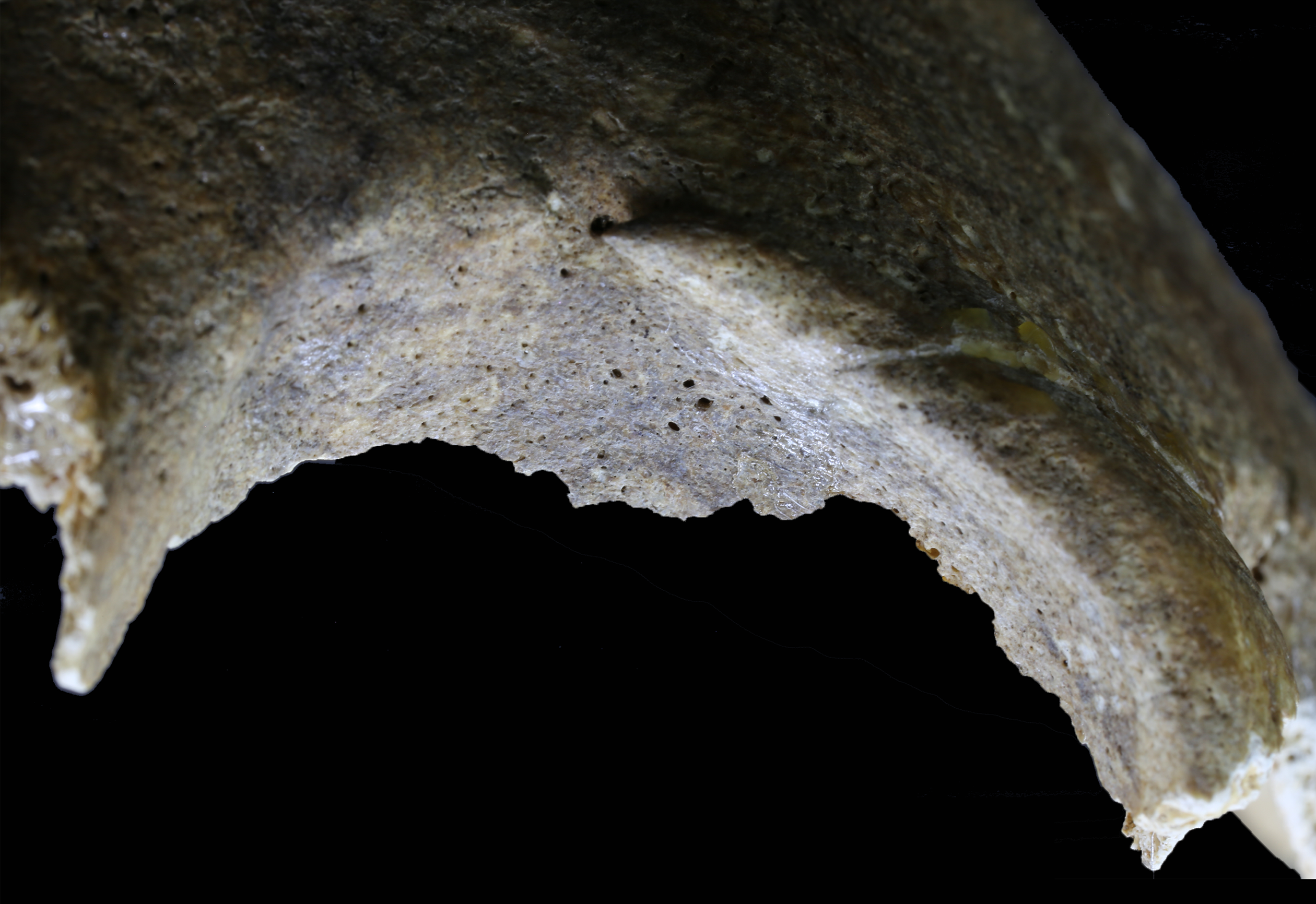

Supplement: S1 Fig — (TIF) [file pone.0140901.s001.tif]

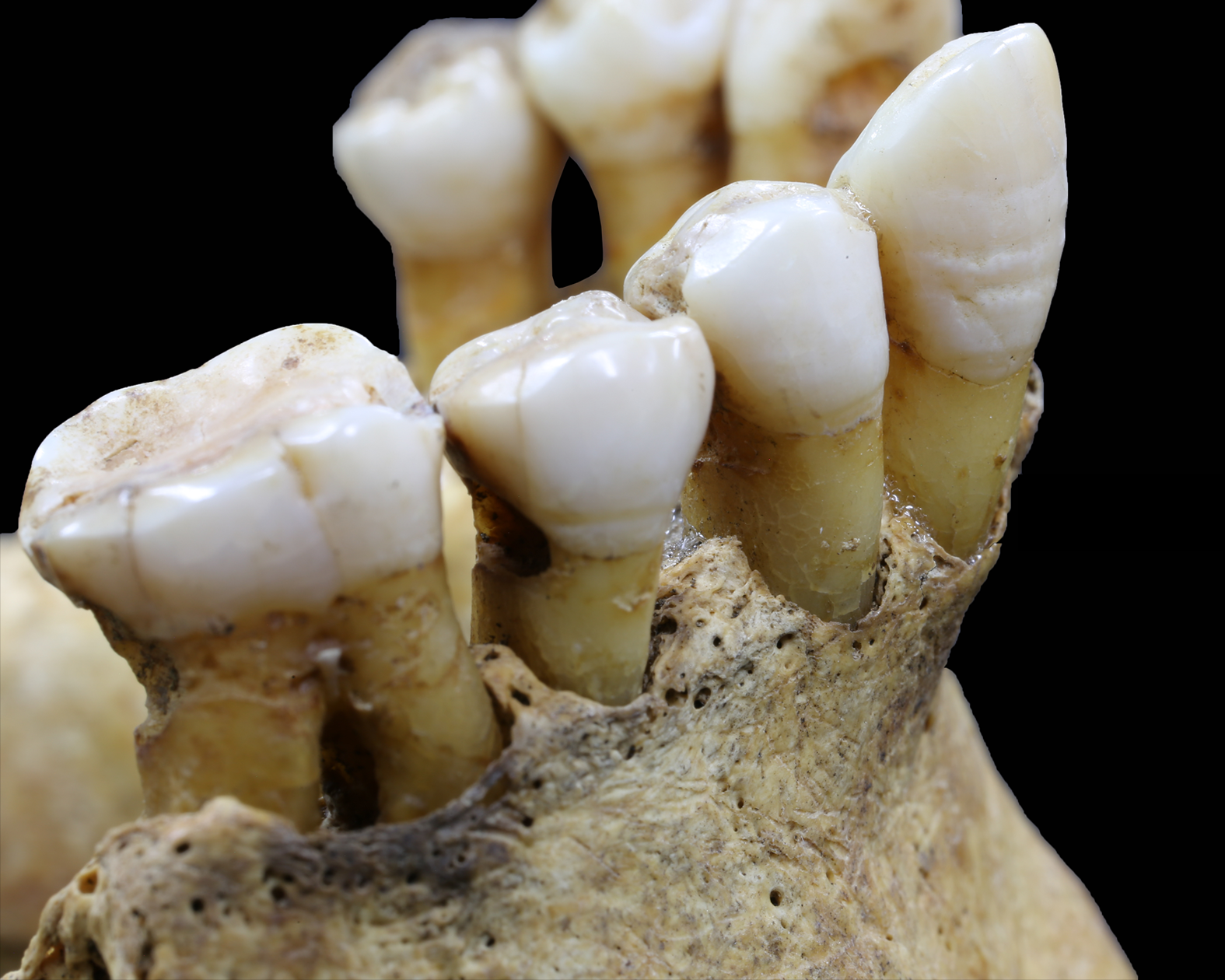

Supplement: S2 Fig — (TIF) [file pone.0140901.s002.tif]
